# Investigation of the Properties of Low Water-to-Solid Ratio Vibro-Press-Formed Alkali-Activated Municipal Solid Waste Incineration Bottom-Ash Concrete

**DOI:** 10.3390/ma18132926

**Published:** 2025-06-20

**Authors:** Gintautas Tamošaitis, Danutė Vaičiukynienė, Diana Bajare

**Affiliations:** 1 Faculty of Architecture and Civil Engineering, Kaunas University of Technology, Studentų st. 48, 51367 Kaunas, Lithuania; danute.vaiciukyniene@ktu.lt; 2 Institute of Sustainable Building Materials and Engineering Systems, Faculty of Civil and Mechanical Engineering, Riga Technical University, 6A Kipsalas, LV-1048 Riga, Latvia; diana.bajare@rtu.lv

**Keywords:** MSWI bottom ash, alkali activation, water-to-solid ratio, vibro-pressing, metakaolin waste

## Abstract

This work focuses on the use of municipal waste incineration bottom ash (MSWI) for the development and production of products suitable for use as construction products. The generation of these ashes is increasing every year due to the incineration of municipal waste. There are currently three incineration plants operating in major cities in Lithuania. The non-hazardous bottom ash remaining from the incineration process is stored in dedicated sorting and aging sites until it is used as an inert form of aggregate for the installation of road foundations. However, it has been observed that these ashes have a tendency to bind and cement when exposed to atmospheric precipitation at the storage site. Based on this characteristic, it was decided in this study to use alkaline activation of the ash to accelerate the bonding process and to create a dense, non-porous composite concrete structure. This activation method is known to create another problem during ash bonding, where the presence of metallic aluminum particles in the ash leads to the release of hydrogen gas and makes the structure of the cured samples porous. For the purposes of the study, it was decided to create a completely different mixture structure and not to use additional water in the mixtures tested. A very low water/solids ratio (W/S) of <0.08 was used for the alkaline activation of the mixtures. All the water required for ash activation was obtained from sodium silicate and sodium hydroxide solution. Metakaolin waste (MKW) was used to adjust the SiO_2_/Na_2_O/Al_2_O_3_ ratio of the mixtures. Vibro-pressing was used to form and increase the density of the samples. And for the formation of the concrete structure, 0/4 fraction sand was used as aggregate. The final alkali-activated sample obtained had properties similar to those of the very widely used vibro-pressed cementitious paving tiles and did not exhibit hydrogen evolution during alkali activation due to the very low W/S ratio. The best results were achieved by samples with a highest compressive strength of 40.0 MPa and a tensile strength of 5.60 MPa, as well as a density of 1950 kg/m^3^. It is believed that this alkaline activation and vibro-pressing method can expand the use of MSWI ash in the development of building products.

## 1. Introduction

Municipal waste is burned in combustion chambers at a high combustion temperature, which is raised by the supply of air. The gases and fly ash from combustion are separated in energy generation and purification systems. However, a large proportion of the waste, around 25%, remains in the combustion chamber. This ash is popularly referred to as bottom ash because of its location in the combustion chamber. It is a mixture of fully incinerated slag and ash of various fractions. The bottom ash is then cooled with water and transported to storage sites by special transport. In the storage sites, it is sorted, cleaned of coarse scrap metal waste and stored until it is used as a base aggregate in road construction. It has been noticed that the ash at the storage site from the incineration plant starts to bind and cement in piles. This creates difficulties for bottom-ash handlers. Although the cementation effect is not strong and largely disappears once the ash has been moved, this characteristic creates a presumption that the bottom ash will continue to cement.

The valorization of municipal solid waste incineration (MSWI) ash is a topical issue. Several studies show that this type of ash can be used as a precursor for alkali-activated binders. In terms of chemical composition, CaO, Al_2_O_3_, SiO_2_ and Fe_2_O_3_ dominated in MSWI ash, and this composition make the ash attractive for the production of alkali-activated materials. Liu et al. [1] found that alkali-activated MSWI ashes reached a compressive strength of 8.8 MPa after 56 days using a 6% Na_2_O alkali activation solution. The main hydration product was C-S-H gel. Maldonado-Alameda et al. [2,3] reported that it is possible to use MSWI ash as the sole precursor of an alkali-activated binder and to achieve a maximum compressive strength of 6.7 MPa. In this case, a complex alkaline activator was prepared from sodium silicate (80% by weight) and NaOH (20% by weight). The NaOH concentration was 6 M. New formed hydration products were calcium silicate hydrate and gehlenite. In another study [4], MSWI ash, as the only precursor, was activated with soluble glass or NaOH solution. It was concluded that soluble glass is a better activator than NaOH solution and the main factor is the fineness of the MSWI ash. In this case, the compressive strength values were in the range of 3–10 MPa.

Most studies on MSWI ash focus on the composite precursor. Due to the low activity of MSWI ash, the precursor is prepared from several components. Initial materials containing a higher content of silicon and aluminum compounds in active form are used together with MSWI ash. The use of MSWI bottom ash alone for the preparation of alkali-activated binder precursors is not recommended by Chen et al. [5]. Such binders have low mechanical properties due to the metallic Al which acted as porous creating agent. Another important reason is the lack of reactive compounds, dominated by CaO, Al_2_O_3_, SiO_2_ and Fe_2_O_3_. In the study [6], the reactive aluminosilicate phase was increased by the addition of metakaolin in the MSWI ash precursor. A geopolymer was produced from the precursors consisting of a large amount of MSWI ash (50–70 wt%) by alkaline activation at room temperature. Tan et al. [7] prepared geopolymer from metakaolin and MSWI ash. The MSWI ash participated in the geopolymerization and modified the geopolymer reaction products. Up to 5 wt% MSWI ash in blends with metakaolin precursor increased strength (from 3.1 to 9.2 MPa after 3 days), but up to 5 wt% the strength decreased significantly. The increase in strength could be attributed to the incorporation of calcium ions into the system, which accelerates the geopolymerization and form addition products such as C-S/A-H. In the results reported by Jin et al. [8], the compressive strength reached 11.97 MPa using triple alumina-silicate initial materials such as MSWI ash, metakaolin and slag as precursors. MSWI ash was the most abundant component in the systems and accounted for 70% by weight of the total precursor components. Liu et al. [9] stated that metakaolin in the blends with MSWI ash up to 10 wt% significantly increased the compressive strength of alkali-activated binders (from 8.04 MPa to 18.08 MPa after 28 days), and the alkali-activated activator was soluble glass. The main hydration products were calcium silicate/aluminum) hydrate and ettringite. Thus, the selection of an optimal composition of MSWI ash precursors and other starting materials with an active form of silicon and aluminum compounds and suitable alkali activator can overcome the negative aspect of these fly ashes.

In alkali-activated binder systems, the combination of low alkali activator content and press molding solves the problems of efflorescence, high water-filled pores, shrinkage on drying, drying-induced cracks [10] and high-level water absorption [11]. In this case, the mechanical properties of the geopolymer are improved compared to molding, even when the curing process is carried out at room temperature [12]. In Shao et al. [13], the mold press technique was used to prepare samples from alkali-activated MSWI ash. After molding, a core-shell structure was formed during the hydration process and the particles of the ash were surrounded by N-A-S-H gel. This pressing-formed technology also improves the strength characteristics. The best alkaline activator was a mixture of sodium hydroxide solution and water glass. A compressive strength of 7.9 MPa was achieved with a forming pressure of 10.2 MPa. Wang et al. [14] prepared a mortar using the pressing method from mixtures of alkali-activated MSWI ash and blast furnace slag with iron tailing sand as fine aggregate. The optimum ratio of MSWI ash to blast furnace slag was 2:8, and MSWI ash was used as alkali activator. The higher forming pressure resulted in higher strength of the samples. The optimum forming pressure of 35 MPa resulted in a compressive strength of about 34 MPa. The positive effect of the forming pressure can be explained by the acceleration of the hydration reactions, the improvement of the microstructure by the increase in the hydration products and the reduction in porosity. Khater et al. [15] combined a low liquid-to-solid ratio with compression molding, and these two factors led to composites with good main properties. A geopolymer composite was formed from a mixture of ground granulated blast-furnace slag and metakaolin activated with NaOH solution. Sand and granite fines were used as an aggregate to produce the composite. In this case, the maximum forming pressure was 50.00 MPa, giving a compressive strength of 35.80 MPa. In another study [16], the geopolymer was prepared without additional alkaline activators, and the alkali was obtained from MSWI fly ash. Pressing techniques were used to increase strength, especially at an early hydration age. It was found that the pressing process closely binds the solid particles and speeds up the geopolymerization reaction.

A new contribution of the research work is the use of non-hazardous bottom ash to produce cement-free composites. This study presents a new method of mixture formation using a very low w/s ratio of <0.08 without the use of additional water, all the water required for activation being provided by a solution of soluble glass and sodium hydroxide. Due to the very low W/S ratio, the vibro-pressing method was used for sample formation. The aim of this work is to investigate the feasibility of using very low water-to-solid ratios in the formulation of alkali-activated municipal solid waste incineration bottom-ash concrete with sand aggregate and admixtures by vibro-pressing.

## 2. Materials and Experimental Techniques

### 2.1. Characterization of Initial Materials

**Figure 1:**
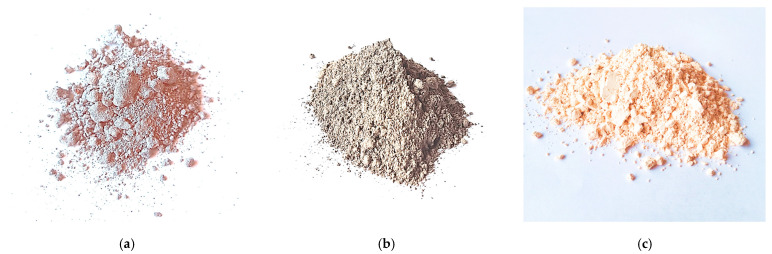
(MSWI) samples of bottom ash before milling (**a**) and after milling (**b**), Metakaolin waste (**c**).

**Figure 2:**
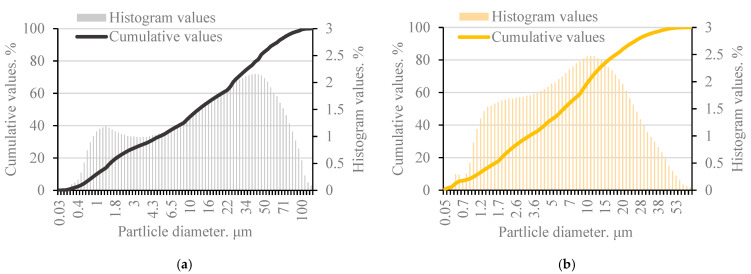
Particle-size distribution of MSWI bottom ash (MSWI) (**a**) and metakaolin waste (MK) (**b**).

During this study, MSWI bottom ash was collected for the production of alkali-activated concrete. This is non-hazardous ash, matured outdoors. However, there is another known problem with the use of this ash. Municipal waste contains all kinds of waste, including various metals, particularly metallic aluminum, which is coated with an oxide film during combustion and remains in the ash. Only large magnetic metallic waste is separated at the storage site, but aluminum cannot be separated. The bottom ash taken for analysis has not undergone any treatment and has been taken in its full composition as stored on the storage site (Figure 1a). In the laboratory, the unsorted bottom ash was dried at 100 °C until the ash mass stopped changing. The dried bottom ash was then milled in two stages, first in a large-volume vibratory mill and then again in a small-volume ball mill, ensuring the highest level of ash milling (Figure 1b). Laser granulometry was used to measure the fineness of the milled bottom ash. The ash bulk density before milling was 1274.0 kg/m^3^ and after milling 1416.0 kg/m^3^ (Figure 2a).

Metakaolin waste. The metakaolin waste used in this study is generated at the plant of UAB “Stikloporas”, Lithuania (Figure 1c). This waste is generated during the production of foamed (porous, blown glass) granules using kaolinite clay powder as an anti-agglomeration agent. The industrial by-product produced is metakaolin waste. The waste metakaolin used is contaminated with foamed glass particles and therefore has a different oxide composition compared to conventional metakaolin. Metakaolin waste has a density of 2540 kg/m^3^ and a specific surface area of 565.3 m^2^/kg (Figure 2b). XRF analysis of MSWI bottom ash and metakaolin waste oxides is shown in Table 1.

**Figure 3:**
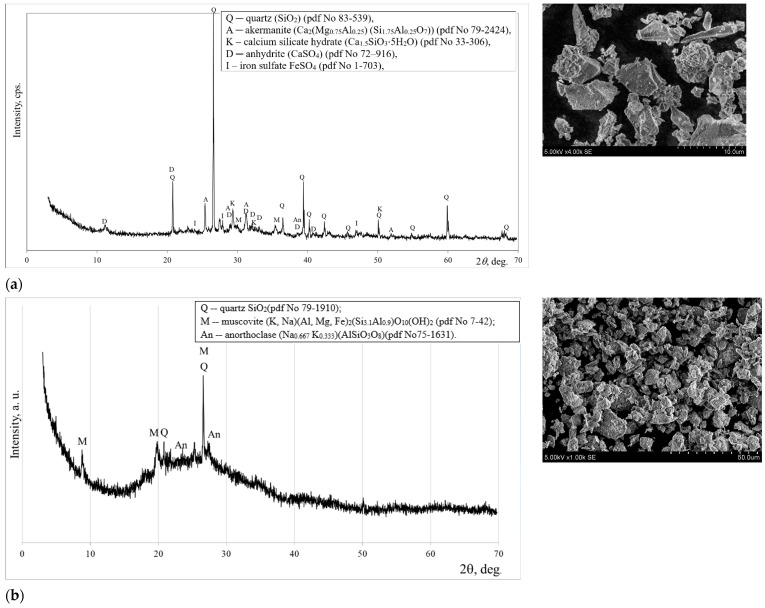
Mineral composition according to XRD and microstructure according to SEM of MSWI bottom ash (**a**) and metakaolin waste (**b**).

The mineral composition of MSWI ash, as determined by XRD analysis, is characterized by the prevalence of quartz and akermanite, with minor traces of C-S-H, anhydrite, and iron sulfate (Figure 3a). A similar mineral composition of MSWI ash was found by Carvalho et al. [17]. Microscopic analysis (SEM) revealed that the particles of ground MSWI ash exhibit irregular shapes with sharp edges (Figure 3a).

Quartz, muscovite and anorthoclase as crystalline compounds are identified in a metakaolin waste sample (Figure 3b). The morphology of the particles of metakaolin waste shows a layered structure. As indicated by earlier studies [18], the existence of platelets and a layered structure has also been identified in the microstructure of metakaolin waste.

**Figure 4:**
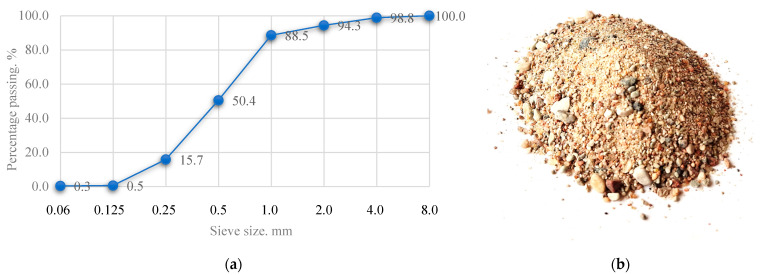
Granulometric composition of sand aggregate (**a**) and sample of the sand (**b**).

Fine aggregate-sand is one of the main components of concrete, which acts as a fine aggregate. The use of sand in alkaline-activated concrete produces a type of concrete that uses only sand as aggregate, without coarse aggregate (e.g., gravel or crushed stone). It is a fine-grained concrete, often used where a smooth surface, thin layers or better workability are required. The sand used is natural, clean and free of clay, silt and organic impurities. The sand may contain moisture, but to ensure that the W/S ratio of the mixture is as low as possible, the sand was dried in the laboratory at 100 °C until the mass of the sand stopped changing. The sand fraction size ranges from 0 to 4 mm. A uniform distribution of sand grains improves the density and strength of the concrete. The granulometric composition of the sand is given in Figure 4.

The alkaline activator was made from a combination of sodium hydroxide and sodium silicate solution. Sodium hydroxide NaOH as a commercial reagent was used in granular form (99% purity, DeltaChem, Ústí nad Labem, Czech Republic). Sodium silicate hydrate solution (Silpur, Wrocław, Poland) had a silicate modulus of 3.0 and ρ = 1290 kg/m^3^. The concentration of the sodium silicate hydrate solution was 50%.

### 2.2. Quantities of Initial Materials for the Preparation of Mixtures

**Figure 5:**
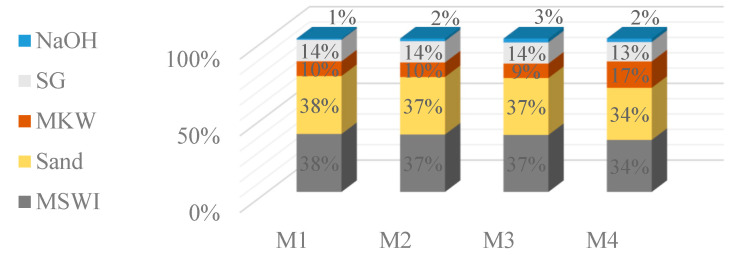
The proportions of the components of the mixtures. Notes: SG—soluble glass, MKW—metakaolin waste, MSWI—municipal solid waste incineration ash.

The mixtures were designed taking into account the water/solid ratio of the mixture, the fineness of the ash and metakaolin waste particles, the gradiometric composition of the sand, the content of the alkaline activator solution and the SiO_2_/Na_2_O/Al_2_O_3_ ratio. The design of the mixtures was based on the use of more than 50% bottom ash in the binder. The parameters and final properties of the mixture and the samples were controlled by the amount and composition of the activator and the metakaolin additive. A fine aggregate, natural sand, was used to increase the mechanical strength of the samples and to ensure the formability conditions of the samples. Under these conditions, the mixtures produced during the test showed suitable conditions for the vibro-pressing and molding of the samples. In this case, the mixtures were first mixed by mixing equal parts of dry sand and bottom ash 50/50. This proportion was used in all mixtures. The proportion of metakaolin waste added to the mix was 12–25% of the total mix. These ingredients form the fine aggregate and binder of the mixture. A solution of sodium hydroxide and sodium silicate was used to activate the binder of the mixture. The solution was prepared by dissolving an appropriate amount of sodium hydroxide granules in a 50% sodium silicate solution, thereby adjusting the Na_2_O content of the activator solution and making it strongly alkaline. This also ensures a good distribution of sodium hydroxide in the volume of the mixture and a good reaction with the binder. The proportions of the components of the mixtures are given in Figure 5.

No additional water was used in the mixtures in order to keep the water/solids ratio as low as possible. The use of additional water in the mixtures allows the metallic aluminum particles to react with the sodium hydroxide solution to produce hydrogen gas. It was therefore decided to use only water from the sodium silicate solution for mixing. The proportion of the activator solution is about 15% by weight of the mixture, and the water/solid ratio does not exceed W/S of < 0.08. This ratio is suitable for the formation of the vibro-pressed samples in order to ensure that they retain their shape after molding. The molar ratio of SiO_2_ to Na_2_O in the alkaline activator was chosen as a starting point for the development of the mixtures. These ratios were determined from the proportions of soluble glass and sodium hydroxide used. A ratio of 1.2 was chosen, as previous studies have shown that this value provides the highest strength of the final product [19]. In this case, the mixtures are varied from 1.6 to 1.07 due to the varying sodium hydroxide content of the mixture. The additional SiO_2_ and Al_2_O_3_ oxides from the metakaolin waste have been taken into account in the calculation of the SiO_2_/Na_2_O/Al_2_O_3_ molar ratios. The compositions of the mixtures are given in Table 2.

**Figure 6:**
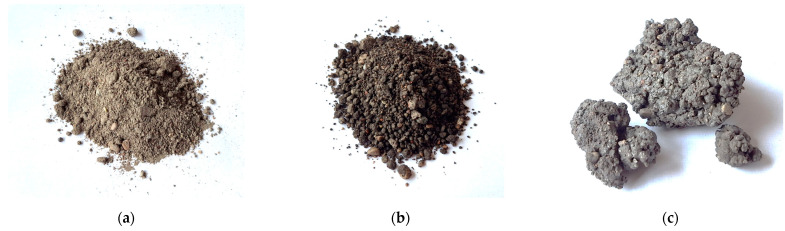
Image of the mixture after mixing the dry parts of the mixture (**a**) with the addition of the activator (**b**), left to rest for one day (**c**).

The dry part of the mixture was fully mixed in the container with an electric high-speed mixer until a homogeneous form was achieved, for about 30 s. After mixing, the activator solution was added to the container and further mixed for 30–60 s until a darkening of the color of the mixture was observed, i.e., the particles of the dry mixture became wet. On further mixing for 30–60 s, the mixture particles became fully wet and started to coalesce. At this stage, it is considered that the mixture has been fully and properly mixed, as it has changed from a sandy and loose consistency to a non-dusty and moist consistency and is suitable for forming. If left undisturbed, this mixture forms loose granular fragments. The final view of the mixture is shown in Figure 6.

**Figure 7:**
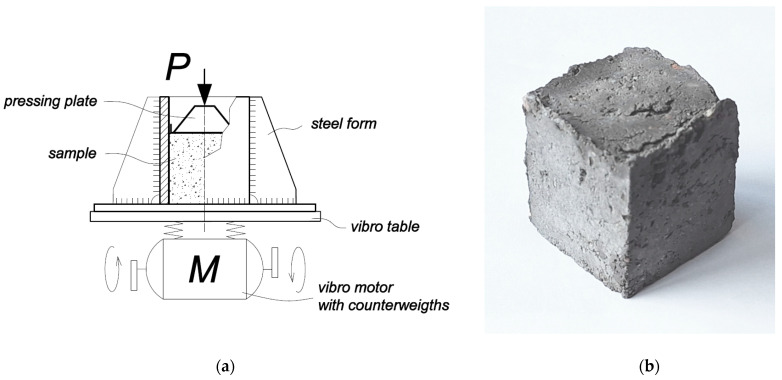
Drawing of the sample forming (**a**) and pressed sample (**b**).

The forming of the samples was carried out in a mold made for this purpose. The form is made of steel sheets welded together to form a semi-collapsible open container which can be filled with the mixture and the mixture compressed in the form. Once fully assembled and restrained, the form filled with the mixture is attached to the vibrating table. During the vibro-pressing, the mixture is simultaneously vibrated on the vibrating table and compressed from above. The calculated pressure during compression of the samples was P = 2.5 MPa. The compression was carried out by pressing the lid of the mold by hand. During the pressing, a sudden compression of the mixture is felt, reaching about 15–25% of the height of the mold and then stopping until it is no longer compressible. The mixture was kept in the form for about 10–15 s during vibro-pressing. Afterwards, the pressing form was disassembled, and the sample was removed from the form. The press form for this study is made in such a way that the final dimensions of the pressed sample are 40 × 40 mm (±0.25–0.5 mm) in cross-sectional area and 55–60 mm in height, depending on the filling of the form. A drawing of the sample forming and a pressed sample are shown in Figure 7.

The prepared pressed samples 40 × 40 × 55–60 mm after molding were placed in a sealed container over water and kept tightly sealed for 7 and 28 days to prevent evaporation of water. The samples were cured at 20 °C throughout the curing period.

### 2.3. Experimental Techniques

Initial materials and samples were analyzed using the following methods.

X-ray fluorescence (XRF) analysis. Applied to study the oxide and/or elemental composition of the initial materials, XRF was used to determine the composition of the constituent oxides. Laboratory equipment: XRF spectrometer Bruker X-ray S8Tiger WD with Rh tube up to 60 eV.

X-ray diffraction (XRD) analysis. The mineral composition of the initial materials was assessed using XRD. The laboratory equipment consisted of a diffractometer “D8 Advance” (Bruker AXS, Karlsruhe, Germany) operating on a 40 kV voltage tube and 40 mA current tube. The X-ray beam was sorted with a 0.02 mm Ni filter to select the CuKα wavelength. Powder X-ray diffraction patterns were identified according to the recommendations of the PDF-2 database.

Laser diffraction. The parameters of the components (density, particle-size distribution from 0.1 to 500 μm and specific surface area) were checked by laser diffraction (dry method). The laboratory equipment was a CILAS 1090 LD laser-scattering particle-size analyzer.

Compressive strength test. The mechanical properties of the alkali-activated concrete were investigated by means of a compressive strength test at 7 and 28 days according to EN 196-1. The concrete samples tested were cubic samples of dimensions 40 × 40 × 40 mm. Laboratory equipment was a Zwick Z100 universal analysis machine (ZwickRoell, Ulm, Germany) with a test speed of 0.6 mm/min.

**Figure 8:**
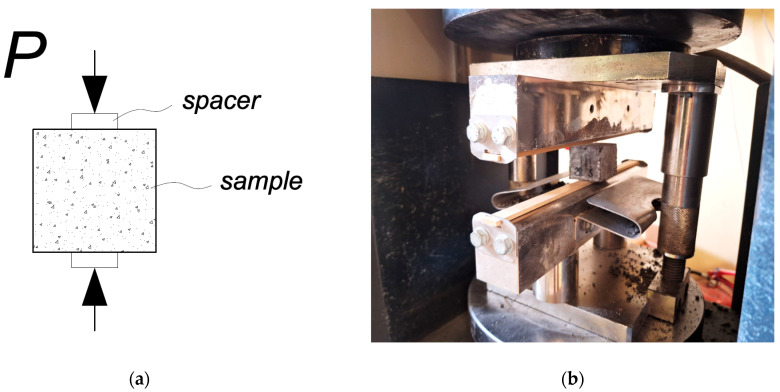
Drawing of the sample tensile splitting test scheme (**a**) and sample testing (**b**).

Tensile splitting test. The mechanical properties of the alkali-activated concrete were investigated by longitudinal splitting strength tests at 7 and 28 days according to EN 1338. The concrete samples tested were cubic in shape, with dimensions of 40 × 40 × 50 mm. Laboratory equipment was a Digmax Plus analysis machine. A drawing of the sample tensile splitting test is shown in Figure 8.

The density of the alkali-activated concrete was determined according to EN 12390-7 [20].

Water absorption and softening coefficient. The water absorption and softening coefficients provide an assessment of durability, water resistance and compaction of alkali-activated concrete. The softening factor is the ratio of the compressive strength of the saturated sample to that of the dry sample, determined by the following Equation (1):

K = C_w_/C_d_ (1)

where C_w_ is the compressive strength in MPa of the samples cured for 28 days and 24 h of soaking in water; C_d_ is the compressive strength in MPa of the samples cured for 28 days and 24 h of drying.

Water absorption is the ratio of water absorbed to the dry mass of the test sample, as given by the following Equation (2):

W_a_ = (M_i_ − M)/M × 100% (2)

where M_i_ is the mass of the sample after saturation with water for 24 h, and M is the mass of the sample after drying to constant weight.

Fourier transform infrared spectroscopy. The mineralogical validity of the AAB synthesis products was verified by Fourier transform infrared spectroscopy (FT-IR). Laboratory equipment was a Perkin Elmer FT-IR System spectrometer (Waltham, MA, USA). One mg of the material was mixed with 200 mg of KBr and compressed in a forming press under vacuum to allow for the infrared analysis.

Scanning electron microscopy. The microstructures of the initial materials and the binder were examined by scanning electron microscopy (SEM). The laboratory equipment was a FEI Quanta 200 FEG high-performance SEM (Hillsboro, OR, USA) with a Schottky field emission gun (FEG). In addition, the chemical composition was analyzed with a Bruker Quad 5040 EDS detector (123 eV).

## 3. Results and Discussion

### 3.1. Physical Properties of Alkali-Activated Concrete

**Figure 9:**
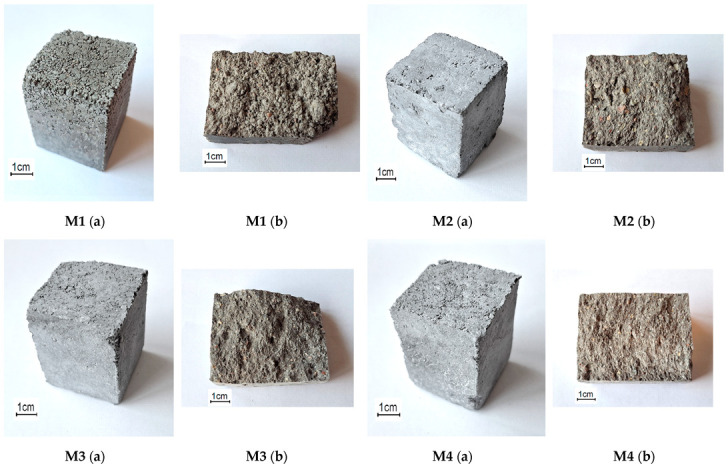
Images of the samples (**a**) and surfaces of samples after the splitting test (**b**) of the corresponding mixture.

The two sample production methods used in the study allowed the creation of different sample structures. Due to the very low W/S ratio of <0.08, no hydrogen release was observed in the samples during alkaline activation. It is known that the alkaline activation method causes a reaction between the metallic aluminum particles in the ash and the sodium hydroxide, which leads to the release of hydrogen gas and forms pores in the mixture, making the structure of the sample more porous. This reduces the durability of the alkali-activated concrete and deteriorates its mechanical properties. The lack of water in this case did not lead to the formation of pores in the mixture, and the mixture did not expand. At the same time, a vibro-pressing method was used to increase the formation and density of the samples, which improved the mechanical properties of the samples. The final alkali-activated sample obtained had properties similar to those of the very widely used vibro-pressed cementitious paving tiles. Images of the samples and the surfaces of samples after the splitting test of the corresponding mixture are shown in Figure 9.

### 3.2. Compressive, Splitting Strength Properties and Density of Alkali-Activated Concrete

**Figure 10:**
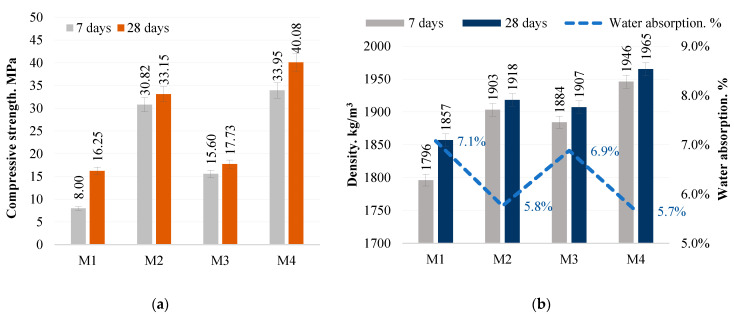
Alkali-activated bottom ash (MSWI) concrete compressive strength (**a**) and density (**b**).

Four mixtures were created for the study—M1, M2, M3, and M4. In the mixtures M1–M3, the sodium hydroxide content was varied. As a result, the compressive strengths and densities of the hardened samples were different. This can be explained by the ratio of SiO_2_/Na_2_O/Al_2_O_3_ in the mixtures. The M1 samples had the lowest sodium hydroxide content. This can be seen from the SiO_2_/Na_2_O ratio of 3.52 and the lower Na_2_O/Al_2_O_3_ ratio of 1.59. The sodium hydroxide content of these samples is believed to be too low for alkaline activation of the bottom ash. The low sodium content does not result in a sufficient amount of binding material and a suitable sample structure. The M2 samples showed the highest compressive and splitting strengths and the highest sample density due to the optimum SiO_2_/Na_2_O/Al_2_O_3_ ratio. The M3 samples showed lower compressive strengths than the M2 samples. This can be explained by the lower SiO_2_/Na_2_O ratio of 2.42 and the higher Na_2_O/Al_2_O_3_ ratio of 2.31. In this case, the SiO_2_ oxide content of the samples was too low, and Al_2_O_3_ oxide was too high. Therefore, the M3 mixture was supplemented with a double proportion of metakaolin waste, the majority of which is SiO_2_. This resulted in M4 samples with a higher SiO_2_/Na_2_O ratio of 3.70 and a lower Na_2_O/Al_2_O_3_ ratio of 1.16. The SiO_2_/Na_2_O/Al_2_O_3_ ratios of these samples are the same as those of the M2 samples, but the higher SiO_2_/Na_2_O ratio and the higher SiO_2_ and lower Al_2_O_3_ content resulted in the highest compressive, splitting and density values (Figure 10). When comparing the results obtained with the studies of other authors, the compressive strength was similar or better after 28 days of hardening. However, it is necessary to take into account that the initial materials used by other authors, including the ash used, were different from those used in this study [21].

**Figure 11:**
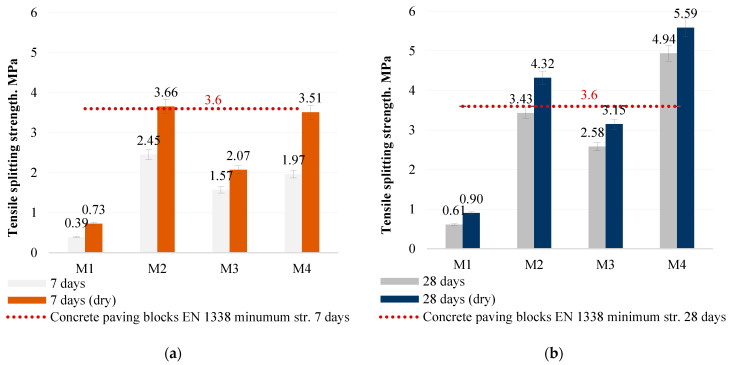
Alkali-activated bottom ash (MSWI) concrete tensile splitting strength after 7 days (**a**) and after 28 days (**b**).

Even the smallest amount of sodium hydroxide in the M1 mixture would result in a porous structure of the sample if water were added to the mixture. However, in this case the density of the samples is twice as high as that of porous lightweight concrete, 800–1000 kg/m3, and is similar to that of normal concrete, 2000 kg/m^3^. In this case, the average density of the samples is 1900 kg/m^3^. No coarse aggregate was used in the mix, as is commonly used in concrete paving. The addition of coarse aggregate to the mix would have resulted in sample densities in excess of 2000 kg/m^3^. Analogues of this type of concrete made with a cementitious binder are commonly used for the installation of concrete pavements. To investigate the material used in the study and its suitability for pavement applications, an additional test, the tensile splitting strength test, was used as the main test for determining the strength of concrete pavements. Santhosh et al. [21] conducted a splitting test on concrete paving blocks containing ash and glass powder, the results of which comply with the standard. Other authors [22] found that the split tensile strength of geopolymer paver blocks made with fly ash and brick kiln rice husk ash after 28 days of curing was similarly lower (3.30 MPa) than that found in this study (5.59 MPa). The minimum limit for the splitting test of 3.6 MPa is applicable to concrete pavers with cementitious binder [23]. Unlike conventional concrete pads, the test samples in this study did not use OPC binder or thermal curing conditions. The results of this test are directly related to, and generally confirm, the compressive strength and density results, but the splitting strength results of the M2 and M4 samples show that the samples reach the minimum test value after 7 days and exceed the minimum test value after 28 days (Figure 11).

### 3.3. Softening Coefficient and Water Absorption Properties

**Figure 12:**
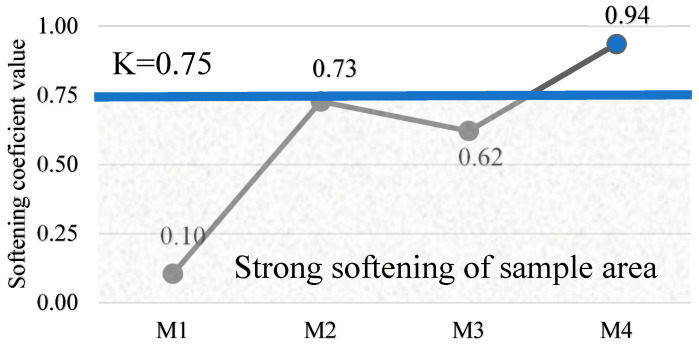
Alkali-activated bottom ash (MSWI) concrete softening coefficient after 28 days.

The results of the softening factor of the specimens suggest that the chosen binder content ensures the durability of the concrete. The specimens were cured for 28 days, dried completely and stored in water for a further 24 h. Their compressive strength was then compared with that of the specimens cured for 28 days. It was observed that the softening coefficient (k) values of the M1 and M3 specimens are lower than the value of 0.50–0.75 for the average softening coefficient of the strength retained after saturation with water, compared to its strength in the dry state. However, samples with a higher density M2 are 0.50–0.75 for medium softening coefficient value, while samples with the highest density M4 are 0.90–1.00 for high water resistance Figure 12.

From the data obtained, it can be concluded that density is closely related to porosity, which are two opposing physical properties. As the density of the concrete increases, the porosity decreases, which slows down the penetration of aggressive materials, ensures the long-term strength and stability of the structure and results in lower water absorption. The low water absorption percentage of concrete is usually an indication of its high density and durability, which is crucial in many applications. In this case, the water absorption of the samples was between 5% and 10%, and the concrete can therefore be considered to be of good quality when assessed on this basis. This result is due to the low water/binder ratio (W/S < 0.08), the vibro-pressurization of the mix and the use of a waterproofing additive, soluble glass.

### 3.4. Microstructure Analysis Results of Concrete

#### 3.4.1. FTIR Spectrum

**Figure 13:**
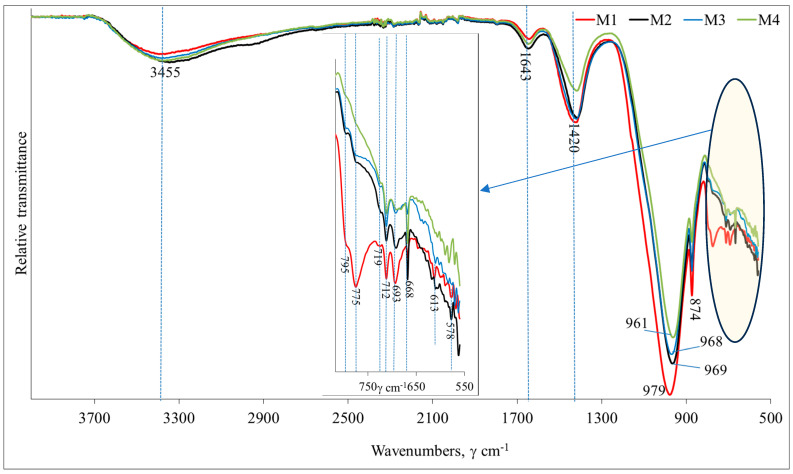
FT-IR curves of vibro-press-formed alkali-activated MSWI bottom-ash concrete. The samples are numbered according to Table 2.

In order to identify the functional groups that are important for assessing the level of geopolymerization of the binders, FT-IR analysis was performed on all four samples (Figure 13). It was detected that all curves have similar characters. The bands detected at 3455 cm^−1^ and 1643 cm^−1^ can be ascribed to the stretching vibration of OH and bending vibration of HOH, respectively [24]. The vibration of carbonate groups (could be CaCO_3_ and Na_2_CO_3_) were assigned to peaks at 712, 874 (the sharp peak) and 1420 cm^−1^ [25]. The intensity of these peaks is similar in the M1, M2 and M3 samples and decreases in the M4 sample. This could be explained by the additional amount of metakaolin waste in the system which led to the incorporation of calcium and sodium in the geopolymerization products. The bands at 775 and 795 cm^−1^ (double peak), 693 cm^−1^ could be attributed to vibrations of Si-O quartz.

The strongest bands are detected in the range of 968–997 cm^−1^ and are attributed to the Si or Al–O stretching vibrations. These bands gradually shift to the side of lower wavenumbers and indicate a higher level of geopolymerization [26]. The transition of amorphous compounds to crystalline ones (for the samples with metakaolin waste: M2, M3 and M4) is confirmed by the shape of the main band, which becomes more narrow and sharp [27]. The increase in the polymerization product calcium led to the product becoming more crystalline, while polymerization decreased and the intensity of peaks gradually decreased for samples M2, M3 and M4 [28].

Thus, the inclusion of metakaolin waste in the mixtures may have led to the formation of additional geopolymerization products, such as gels, which transform into zeolitic crystalline compounds, from additional calcium and sodium compounds. These newly formed amorphous and crystalline geopolymerization products had a positive effect on the mechanical properties of the samples.

#### 3.4.2. Surface Quality Analysis of Alkali-Activated Concrete

**Figure 14:**
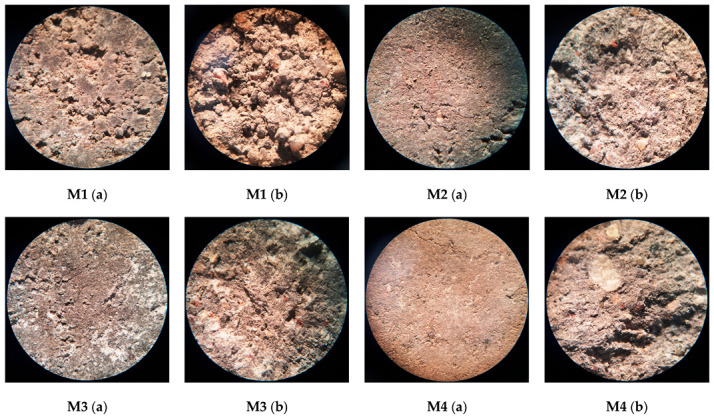
Microscopic ×10 magnification of the surface of the samples (**a**) and the surface after the splitting test (**b**) of the corresponding mixture.

Optical microscopy was used for the evaluation of the microstructure of the investigated samples (Figure 14a,b). M1 and M3 samples showed the comparable porous structure both before and after splitting tests. This porous microstructure resulted in relatively low strength values and high softening coefficients. The structure of the other remaining samples (M2 and M4) was more compact compared to the M1 sample, and this resulted in higher strength values. Sample M3 also showed a white precipitation of material both before and after the splitting test, and this is likely to be uncured alkaline activator released on the surface of the sample. It is likely that this white precipitate is the cause of the higher softening coefficient (0.62) for the M3 sample.

**Figure 15:**
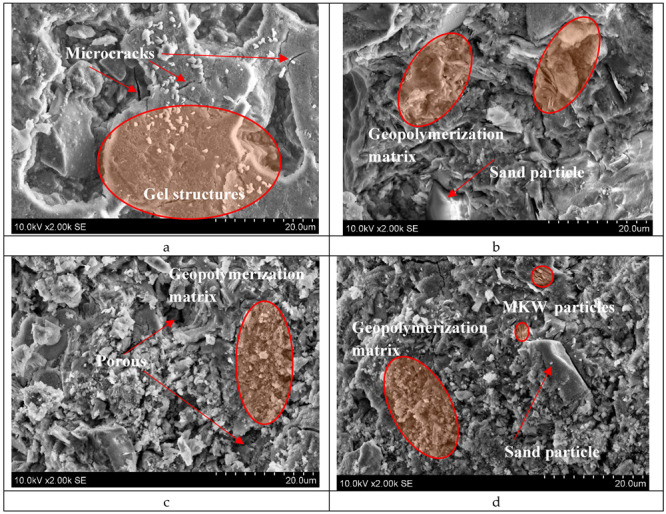
Microstructure according to SEM of vibro-press formed alkali-activated MSWI bottom-ash concrete: (**a**) M1, (**b**) M2, (**c**) M3, (**d**) M4. The samples are numbered according to Table 2.

The microstructure of the M1 sample is dominated by gel-like structures (Figure 15a). The highest gel content was confirmed by FT-IR analysis. The strength values were not high (16.25 MPa was reached after 28 days of curing), which could be due to microcracking caused by the drying process [29]. The gel structures are susceptible to drying, and for that reason some micro-cracks were found in the dense matrix.

When in the M2 samples the ratio SiO_2_/Na_2_O decreased from 3.52 to 2.87 (Table 2), a more compact microstructure was detected (Figure 15b), which corresponds to high values of strength. A molar ratio of SiO_2_/Na_2_O = 2.42 resulted in a more porous microstructure, which corresponds to a significant reduction in the strength properties (Figure 15c). In sample M4, the SiO_2_/Na_2_O molar ratio was 3.70, which is consistent with a compact microstructure (Figure 15d). The quartz particle was embedded in a matrix of geopolymerization products. Only a small amount of layered metakaolin waste structures were found in this sample. The hydration product resulted in a compact microstructure combined with high mechanical properties.

## 4. Conclusions

The results show that the low water/binder ratio and the vibro-pressing method did not lead to the formation of pores during alkaline activation. This allowed the production of dense samples with good mechanical properties similar to traditional vibro-pressed cementitious tiles.

The study showed that the optimum SiO_2_/Na_2_O/Al_2_O_3_ ratios, as in the M4 mix, directly contribute to the best mechanical properties and to the formation of a dense concrete structure. The M4 mix had the highest compressive strength of 40.0 MPa and a high tensile splitting strength of 5.60 MPa, as well as a density of 1950 kg/m^3^. These properties indicate that the appropriate amount of sodium hydroxide and the SiO_2_-rich metakaolin waste ensure the effective formation of the bonding material, which determines the strength, resistance and durability of the structure.

The study confirms that the density of concrete is closely related to its porosity, the two properties being inversely related: as density increases, porosity decreases. This leads directly to the infiltration of aggressive substances, water and humidity into the concrete structure, so that a higher density contributes to the long-term strength, stability and corrosion resistance of the structure.

Tests on samples have shown that even without the use of additional water in the mix, only a soluble glass solution, thus reducing the water/binder ratio (W/S < 0.08), it is possible to achieve a low percentage of water absorption (5–10%), which is indicative of good concrete quality.

In view of these results, the concretes tested are suitable for paving applications as they provide sufficient mechanical strength, durability and resistance to environmental influences by using alkaline-activated MSWI bottom ash as a binder. These characteristics make this type of concrete a reliable and cost-effective solution for a wide range of applications where durability and resistance to moisture are important.

## Figures and Tables

**Table 1:** Oxide composition of initial material (MSWI ash and metakaolin waste) according XRF, wt%.

Oxides	SiO_2_	CaO	Al_2_O_3_	Fe_2_O_3_	MgO	K_2_O	P_2_O_5_	SO_3_	Cl	TiO_2_	Na_2_O	Other
**MSWI ash**	42.6	33.62	7.82	3.12	3.37	1.59	1.84	3.17	0.35	1.05	0.50	0.97
**Metakaolin waste**	54.28	2.99	31.74	0.91	0.88	0.69	0.10	0.14	-	0.53	7.46	0.28

**Table 2:** Composition of mixtures and quantities of initial materials.

Mix	MSWI Ash	MKW Additive	Alkali Activator			Sand Aggregate	W/S	Molar Ratios		
			Soluble Glass	NaOH	Molar Ratios SiO_2_/Na_2_O			SiO_2_/ Na_2_O	SiO_2_/ Al_2_O_3_	Na_2_O/ Al_2_O_3_
M1	650	165	240	15	1.6	650	0.076	3.52	5.59	1.59
M2	650	165	240	30	1.3	650	0.076	2.87	5.59	1.95
M3	650	165	240	45	1.07	650	0.076	2.42	5.59	2.31
M4	650	330	240	45	1.07	650	0.068	3.70	4.28	1.16

Note: Mix proportions (kg/m^3^), molar ratios (mol/mol).

## Data Availability

The original contributions presented in the study are included in the article, further inquiries can be directed to the corresponding author.
